# The association of active and secondhand smoking with oral health in adults: Japan public health center-based study

**DOI:** 10.1186/s12971-015-0047-6

**Published:** 2015-07-29

**Authors:** Masayuki Ueno, Satoko Ohara, Norie Sawada, Manami Inoue, Shoichiro Tsugane, Yoko Kawaguchi

**Affiliations:** Department of Oral Health Promotion, Graduate School of Medical and Dental Sciences, Tokyo Medical and Dental University, Tokyo, Japan; Department of Comprehensive Oral Health Care, Faculty of Dentistry, Tokyo Medical and Dental University, Tokyo, Japan; Epidemiology and Prevention Division, Research Center for Cancer Prevention and Screening, National Cancer Center, Tokyo, Japan; AXA Department of Health and Human Security, Graduate School of Medicine, University of Tokyo, Tokyo, Japan

**Keywords:** Active smoking, Secondhand smoking, Periodontal disease, Dentition, Functional tooth units

## Abstract

**Background:**

Smoking is one of the major risk factors for oral diseases, and many studies have found that active smoking is closely associated with the prevalence or severity of periodontal disease and fewer remaining teeth. In contrast to the established association between active smoking and oral health, there have been very few studies investigating the effects of secondhand smoking on oral health, and whether secondhand smoking deteriorates oral health has not been fully clarified. The purpose of the present study was to examine whether active and secondhand smoking were associated with the prevalence of severe periodontal disease and number of teeth among Japanese adults.

**Methods:**

Subjects were 1,164 dentate adults aged 55–75 years as of May 2005 who participated in both the Japan Public Health Center-Based Study Cohort I in 1990 and a dental survey in 2005. The dental survey was implemented in the Yokote health center jurisdiction, Akita Prefecture. Participating subjects completed a self-administered questionnaire and a clinical oral examination. The association of smoking status with prevalence of periodontal disease was analyzed using a logistic regression, and with number of teeth or functional tooth units of natural teeth (n-FTUs) using a generalized linear model.

**Results:**

After adjusting for age, education level, history of diabetes, BMI, alcohol consumption, perceived mental stress, presence of a family dentist, and oral hygiene, the odds ratio (OR) of risk for periodontal disease in male subjects was significantly increased in non-smokers with secondhand smoking only at home (OR = 3.14, 95 % CI: 1.08−9.12, *p* = 0.036), non-smokers with secondhand smoking both at home and other places (OR = 3.61, 95 % CI: 1.33−9.81, *p* = 0.012) and current smokers (OR = 3.31, 95 % CI: 1.54−7.08, *p* = 0.002), compared to non-smokers without secondhand smoking. Further in men, current smokers had significantly fewer numbers of teeth (19.7 ± 6.82) and n-FTUs (4.92 ± 4.12) than non-smokers without secondhand smoking (22.2 ± 6.92, *p* = 0.014 and 6.56 ± 4.18, *p* = 0.007). Such significant relationships of smoking status with periodontal disease and dentition were not observed in women.

**Conclusions:**

The present study indicates that active smoking as well as secondhand smoking may have harmful effects on periodontal health in men. Therefore, it is imperative for health and oral health professionals to enlighten people about the negative influence of smoking, not only on their own health but also on others’ health.

## Background

Many clinical, epidemiological and biological studies have demonstrated that not only active smoking but also exposure to other people’s cigarette smoke (secondhand smoking, also called involuntary smoking or environmental tobacco smoking) are associated with detrimental health effects such as asthma, lung cancer and cardiovascular diseases [1, 2]. Smoking is also one of the major risk factors for oral diseases such as periodontal disease and tooth loss, and many studies have found that active smoking is closely associated with the prevalence or severity of periodontal disease and fewer remaining teeth [3–7]. Further, increasing evidence shows that secondhand smoking may aggravate periodontal disease in non-smokers [8, 9].

A study using data from the Third National Health and Nutrition Examination Survey (NHANES III) in the USA reported that the risk of periodontal disease was 1.6 times higher for people with secondhand smoking than for those without [10]. A study with newer 1999–2004 NHANES data also showed that periodontal disease in non-smokers was negatively impacted by secondhand smoking [11]. Another study in the USA demonstrated the risk for severe periodontal disease was increased by 29 % among people exposed to secondhand smoke 1 to 25 h/week; for those exposed 26 h/week, the risk was twice as high as for those who were not exposed [12].

A Japanese study employing the salivary cotinine level to classify subjects into active (cotinine≧8 ng/mL) and secondhand (cotinine 1–7 ng/mL) smokers showed that both active and secondhand smoking increased the odds ratio (OR) for the prevalence of periodontal disease by 4.9 and 2.9, respectively [13]. Another 2-year follow-up study in Japan using salivary inflammatory and microbiological markers found significantly higher risk of having periodontal disease in both active (OR = 2.3) and secondhand (OR = 2.2) smokers compared to nonsmokers [14].

However, in contrast to the established association between active smoking and oral health, very few studies have investigated the effects of secondhand smoking on oral health, and whether secondhand smoking deteriorates oral health has not been fully clarified. A recently published systematic review also concluded that the association between secondhand smoking and periodontal disease remains debatable and requires further investigation, because more rigorous, longitudinal analysis adjusting confounders like daily oral hygiene maintenance protocols is necessary to confirm the association [9]. Further, no studies have been conducted so far of the relationship between secondhand smoking and tooth loss. Therefore, the purpose of the present study was to examine whether active and secondhand smoking were associated with the prevalence of severe periodontal disease and number of teeth among Japanese adults.

## Methods

### Subjects

To prospectively monitor the morbidity and mortality of diseases such as cancer and cardiovascular disorders in a large population-based Japanese sample, the Japan Public Health Center-Based (JPHC) Study Cohort I started in 1990. As a part of the JPHC Study Cohort I, a dental survey was implemented in the Yokote health center jurisdiction, Akita Prefecture in 2005. Recruitment of subjects was carried out by sending invitation letters explaining the purposes and procedures of the dental survey to 15,782 adults aged 55–75 years as of May, 2005, who had participated in the JPHC Study Cohort I. From July 2005 through January 2006, 1,518 subjects completed a self-administered oral health questionnaire and underwent a clinical oral examination. Among participating subjects, 1,164 dentate adults were used for the current analysis after excluding those with missing data for the study variables.

Approval for this study was obtained from the Ethics Committee of the National Cancer Center in Tokyo and the Tokyo Medical and Dental University Ethical Committee, Japan (Approval No. 833).

### Socio-demographics and health related information

Information about socio-demographics (gender, age and education level) and health related information (presence or absence of diabetes history, BMI, alcohol consumption and perceived mental stress) was collected from a self-administered questionnaire conducted in 1990 for the JPHC Study Cohort I. The education level was divided into ‘low (junior high school)’, ‘middle (senior high school)’ and ‘high (any college or higher education)’. The BMI was calculated using the formula [weight (kg)/height (m)^2^]. Alcohol consumption was categorized into ‘nondrinkers or former drinkers’, ‘less than weekly', ‘<150 g/week’, ‘150−299 g/week’, ‘300−449 g/week’ and ‘≧450 g/week’, and perceived mental stress into ‘low', ‘moderate’ and ‘high’.

### Active and secondhand smoking status

A self-administered questionnaire in 1990 inquired about active and secondhand smoking status.

Questions on secondhand smoking collected information about two experiences of secondhand smoking; 1) ‘Have you ever lived with any regular smokers at home for more than 10 years?’ (‘Yes’ or ‘No’), and 2) ‘In any places outside the home, for example at your workplace, how often have you been exposed to secondhand smoke ≧1 h/day?’ (‘almost never’, ‘1 to 3 days/month', ‘1 to 4 days/week’ and ‘almost every day’). Secondhand smoking was defined if a subject answered ‘yes’ to the first question or ‘almost every day’ to the second question. Smoking status was then categorized into six levels: ‘non-smoker without secondhand smoking', ‘non-smoker with secondhand smoking only at home', ‘non-smoker with secondhand smoking only at other places outside the home’, ‘non-smoker with secondhand smoking both at home and other places', ‘past smoker’ and ‘current smoker’.

A 10-year cut off for exposure to secondhand smoke was chosen for the sake of convenience, because at least 10 years and more exposure to secondhand smoke had often been used to investigate the impact of secondhand smoking on the health [15, 16]. Therefore, there was no substantial difference of effects between 9.9 years of exposure and 10.1 years of exposure to secondhand smoke.

### Oral health related information

A self-administered oral health questionnaire inquired about the presence or absence of a family dentist, and a standardized clinical oral examination (excluding third molars) assessed the periodontal, dentition and oral hygiene conditions of subjects in 2005. The clinical oral examinations were performed by 43 participating dentists who were trained based on World Health Organization (WHO) guidelines [17] prior to the examination.

For a periodontal examination, periodontal pocket depths were measured circumferentially for all natural teeth and the deepest pocket depth was recorded for each tooth. Periodontitis was defined when a subject had at least one tooth with pocket depth of 6 mm or greater. Its condition approximately corresponds to the code 4 of Community Periodontal Index by the WHO, which defines a pocket depth of 6 mm or more as severe periodontitis [6]. With regard to dentition status, in addition to the number of teeth, functional tooth units of natural teeth (n-FTUs) [18] was calculated. FTUs is an index of posterior occlusion, which ranges from 0 to 12, with two opposing premolars counted as 1 and two opposing molars as 2.

Oral hygiene of teeth or dentures was visually evaluated by inspecting all teeth or dentures. The scores were: 1) good = plaque covering less than one-third of tooth surfaces; 2) fair = plaque covering more than one-third but less than two-thirds of tooth surfaces; and 3) poor = plaque covering more than two-thirds of tooth surfaces. The person’s worst score was recorded. Inter- or intra- reliability tests among participating dentists were not calculated in the dental survey.

### Statistical analysis

ANOVA was used for testing the difference of mean age and BMI between smoking statuses, and chi-square test for the relationship of smoking status with categorical values such as education level, history of diabetes, alcohol consumption, perceived mental health, presence of family dentist and oral hygiene. The association of smoking status with prevalence of periodontal disease was analyzed using a logistic regression and with number of teeth or n-FTUs using a generalized linear model adjusted for age as well as education level, history of diabetes, BMI, alcohol consumption, perceived mental stress, presence of a family dentist and oral hygiene. Because of a large difference in smoking prevalence between men and women, analyses were conducted by gender. All analyses were performed using IBM® SPSS® 21 J software (IBM Japan Ltd., Tokyo, Japan).

## Results

### Socio-demographics, health and oral health related information

The mean age of all subjects as of 2005 was 65.1 ± 5.75 years. Proportions of non-smokers, past smokers and current smokers were 28.8 %, 28.6 % and 42.6 % in men, and 96.2 %, 1.3 % and 2.5 % in women, respectively (Tables 1 and 2). Among the non-smokers, the numbers of those without and with secondhand smoking were 59 and 100 in men, and 131 and 458 in women, respectively. The numbers of non-smokers with secondhand smoking by place were as follows: only at home, 30 in men and 328 in women; only at other places, 33 in men and 19 in women; and both at home and other places, 37 in men and 111 in women. There were no significant differences in mean age by smoking status in men, but non-smokers without secondhand smoking were significantly younger than non-smokers with secondhand smoking both at home and other places, past smokers, and current smokers in women (*p* = 0.028).

**Table 1:** Characteristics according to active and secondhand smoking status in men

	Non-smoker				Past smoker	Current smoker	*P* value
	Without secondhand smoking	With secondhand smoking					
		Home	Other places	Home and other places			
	(*n* = 59)	(*n* = 30)	(*n* = 33)	(*n* = 37)	(*n* = 158)	(*n* = 235)	
Age, mean (SD)	66.4 (5.23)	66.4 (4.94)	65.5 (4.69)	65.7 (5.03)	65.6 (6.34)	64.7 (5.85)	0.309
Education level, n (%)							
Low	21 (35.6)	11 (36.7)	10 (30.3)	6 (16.2)	41 (25.9)	72 (30.6)	0.029
Middle	33 (55.9)	19 (63.3)	15 (45.5)	25 (67.6)	79 (50.0)	114 (48.5)	
High	56 (8.5)	0 (0)	8 (24.2)	6 (16.2)	38 (24.1)	49 (20.9)	
History of diabetes, n (%)							
Yes	1 (1.7)	2 (6.7)	1 (3.0)	3 (8.1)	3 (1.9)	11 (4.7)	0.372
No	58 (98.3)	28 (93.3)	32 (97.0)	34 (91.9)	155 (98.1)	224 (95.4)	
BMI, mean (SD)	23.2 (2.28)	23.8 (2.74)	23.0 (2.55)	24.0 (3.37)	23.5 (2.38)	23.2 (2.64)	0.377
Alcohol consumption, n (%)							
Non or former drinkers	11 (18.6)	0 (0)	4 (12.1)	6 (16.2)	15 (9.5)	24 (10.2)	0.007
Less than weekly	8 (13.6)	0 (0)	2 (6.1)	1 (2.7)	8 (5.1)	9 (3.8)	
<150 g/week	4 (6.8)	2 (6.7)	3 (9.1)	10 (27.0)	17 (10.8)	22 (9.4)	
150-299 g/week	8 (13.6)	5 (16.7)	5 (15.2)	4 (10.8)	25 (15.8)	31 (13.2)	
300-449 g/week	6 (10.2)	11 (36.7)	5 (15.2)	1 (2.7)	28 (17.1)	41 (17.4)	
>450 g/week	22 (37.3)	12 (40.0)	14 (42.4)	15 (40.5)	65 (41.1)	108 (46.0)	
Perceived mental stress, n (%)							
Low	5 (8.5)	1 (3.3)	1 (3.0)	4 (10.8)	15 (9.5)	20 (8.5)	0.779
Moderate	40 (67.8)	21 (70.0)	20 (60.6)	22 (59.5)	88 (55.7)	138 (58.7)	
High	14 (23.7)	8 (26.7)	12 (36.4)	11 (29.7)	55 (34.8)	77 (32.8)	
Family dentist, n (%)							
Yes	49 (83.1)	28 (93.3)	30 (90.9)	31 (83.8)	135 (85.4)	203 (86.4)	0.752
No	10 (16.9)	2 (6.7)	3 (9.1)	6 (16.2)	23 (14.6)	32 (13.6)	
Oral hygiene, n (%)							
Good	5 (8.5)	5 (16.7)	6 (18.2)	3 (8.1)	24 (15.2)	22 (9.4)	0.534
Fair	38 (64.4)	18 (60.0)	22 (66.7)	27 (73.0)	96 (60.8)	148 (63.0)	
Poor	16 (27.1)	7 (23.3)	5 (15.2)	7 (18.9)	38 (24.1)	65 (27.7)	

Combined cells for proper application of chi-square test

**Table 2:** Characteristics according to active and secondhand smoking status in women

	Non-smoker				Past smoker	Current smoker	*P* value
	Without secondhand smoking	With secondhand smoking					
		Home	Other places	Home and other places			
	(*n* = 131)	(*n* = 328)	(*n* = 19)	(*n* = 111)	(*n* = 8)	(*n* = 15)	
Age, mean (SD)	65.9 (5.10)	65.0 (5.98)	64.4 (6.18)	64.0 (5.45)	61.4 (5.01)	62.7 (5.37)	0.028
Education level, n (%)							
Low	59 (45.0)	112 (34.2)	4 (21.1)	27 (24.3)	1 (12.5)	3 (20.0)	0.001
Middle	51 (39.0)	174 (53.0)	10 (52.6)	62 (55.9)	2 (25.0)	9 (60.0)	
High	21 (16.0)	42 (12.8)	5 (26.3)	22 (19.8)	5 (62.5)	3 (20.0)	
History of diabetes, n (%)							
Yes	2 (1.5)	4 (1.2)	1 (5.3)	0 (0.0)	0 (0.0)	0 (0.0)	0.381
No	129 (98.5)	324 (98.8)	18 (94.7)	111 (100)	8 (100)	15 (100)	
BMI, mean (SD)	22.9 (2.61)	22.7 (2.87)	22.3 (2.78)	23.2 (2.99)	23.0 (1.59)	22.7 (2.74)	0.740
Alcohol consumption, n (%)							
Non or former drinkers	85 (64.9)	241 (73.5)	13 (68.4)	81 (73.0)	5 (62.5)	6 (40.0)	0.205
Less than weekly	21 (16.0)	41 (12.5)	3 (15.8)	10 (9.0)	1 (12.5)	3 (20.0)	
<150 g/week	14 (10.7)	27 (8.2)	1 (5.3)	10 (9.0)	1 (12.5)	3 (20.0)	
150-299 g/week	5 (3.8)	9 (2.7)	1 (5.3)	4 (3.6)	0 (0.0)	2 (13.3)	
300-449 g/week	4 (3.1)	7 (2.1)	0 (0.0)	5 (4.5)	1 (12.5)	0 (0.0)	
>450 g/week	2 (1.5)	3 (0.9)	1 (5.3)	1 (0.9)	0 (0.0)	1 (6.7)	
Perceived mental stress, n (%)							
Low	16 (12.2)	34 (10.4)	0 (0.0)	12 (10.8)	1 (12.5)	2 (13.3)	0.237
Moderate	90 (68.7)	216 (65.8)	12 (63.2)	64 (57.7)	3 (37.5)	10 (66.7)	
High	25 (19.1)	78 (23.8)	7 (36.8)	35 (31.5)	4 (50.0)	3 (20.0)	
Family dentist, n (%)							
Yes	121 (92.4)	299 (91.2)	17 (89.5)	97 (87.4)	7 (87.5)	12 (80.0)	0.405
No	10 (7.6)	29 (8.8)	2 (10.5)	14 (12.6)	1 (12.5)	3 (20.0)	
Oral hygiene, n (%)							
Good	17 (13.0)	45 (13.7)	3 (15.8)	20 (18.0)	2 (25.0)	2 (13.3)	0.715
Fair	93 (71.0)	222 (67.7)	12 (63.2)	76 (68.5)	5 (62.5)	8 (53.3)	
Poor	21 (16.0)	61 (18.6)	4 (21.0)	15 (13.5)	1 (12.5)	5 (33.3)	

Combined cells for proper application of chi-square test

Significant distributional differences were observed in education level in both men and women (*p* = 0.029 and *p* = 0.001) and alcohol consumption (*p* = 0.007) in men by smoking status. The proportions of non-smokers without secondhand smoking were low in subjects with a high education level in men and high in those with a low education in women. The proportion of current smokers was high in subjects with alcohol consumption of >450 g/week in men.

History of diabetes, BMI, perceived mental stress, presence of family dentist and oral hygiene did not have significant relationships with smoking status.

### Association between smoking status and prevalence of periodontal disease

The prevalence of periodontal disease according to the smoking status is shown in Table 3. When adjusting for age in men, the OR for having periodontal disease was significantly higher in non-smokers with secondhand smoking only at home (OR = 2.84, *p* = 0.042), non-smokers with secondhand smoking both at home and other places (OR = 2.70, *p* = 0.042) and current smokers (OR = 3.11, *p* = 0.002) than non-smokers without secondhand smoking. After further adjusting for education level, history of diabetes, BMI, alcohol consumption, perceived mental stress, presence of a family dentist, and oral hygiene, in addition to age, the OR of risk for periodontal disease was significantly increased in non-smokers with secondhand smoking only at home (OR = 3.14, *p* = 0.036), non-smokers with secondhand smoking both at home and other places (OR = 3.61, *p* = 0.012) and current smokers (OR = 3.31, *p* = 0.002) compared to non-smokers without secondhand smoking.

**Table 3:** Prevalence of periodontal disease^a^ according to active and secondhand smoking status

	Non-smoker				Past smoker	Current smoker
	Without secondhand smoking	With secondhand smoking				
		Home	Other places	Home and other places		
Men						
% (No. of cases/subjects)	16.9 (10/59)	27.0 (11/30)	18.2 (6/33)	35.1 (13/37)	24.1 (38/158)	37.9 (89/235)
Adjusted OR^b^ (95 % CI)	1	2.84 (1.04-7.78)	1.11 (0.36-3.40)	2.70 (1.04-7.05)	1.58 (0.73-3.42)	3.11 (1.49-6.47)
Adjusted OR^c^ (95 % CI)	1	3.14 (1.08-9.12)	1.31 (0.41-4.17)	3.61 (1.33-9.81)	1.81 (0.81-4.03)	3.31 (1.54-7.08)
Women						
% (No. of cases/subjects)	23.7 (31/131)	17.7 (58/328)	15.8 (3/19)	21.6 (24/111)	25.0 (2/8)	40.0 (6/15)
Adjusted OR^b^ (95 % CI)	1	0.70 (0.43-1.14)	0.61 (0.17-2.24)	0.90 (0.49-1.66)	1.11 (0.21-5.80)	2.19 (0.72-6.69)
Adjusted OR^c^ (95 % CI)	1	0.66 (0.40-1.10)	0.55 (0.14-2.11)	0.88 (0.46-1.65)	1.03 (0.18-5.85)	1.98 (0.62-6.35)

^a^ ≥ 1 sites with periodontal pocket ≥6 mm

^b^Adjusted for age

^c^Adjusted for age, education level, history of diabetes, BMI, alcohol consumption, perceived mental stress, presence of family dentist, and oral hygiene

As for women, the OR for having periodontal disease in non-smokers with secondhand smoking ranged from 0.55 to 0.90. The ORs in past smokers or current smokers were 1.11 and 1.03, or 2.19 and 1.98, respectively. No significant findings were detected in women.

### Association between smoking status and dentition

After adjusting for age in men, current smokers (19.6 ± 7.02) had significantly fewer teeth compared to non-smokers without secondhand smoking (21.8 ± 7.02, *p* = 0.030) (Table 4). After further adjusting for the other variables besides age, a similar result was obtained. Current smokers (19.7 ± 6.82) had significantly fewer teeth than non-smokers without secondhand smoking (22.2 ± 6.92, *p* = 0.014). No significant differences in the number of teeth were found between other smoking statuses. Findings were similar for n-FTUs. The number of n-FTUs after adjusting for age in current smokers (4.82 ± 4.25) was significantly lower compared to non-smokers without secondhand smoking (6.34 ± 4.25, *p* = 0.014). After adjusting for the other variables along with age, current smokers (4.92 ± 4.12) had significantly fewer n-FTUs than non-smokers without secondhand smoking (6.56 ± 4.18, *p* = 0.007). There were no significant differences in the number of n-FTUs between other smoking statuses.

**Table 4:** Number of teeth and n-FTUs according to active and secondhand smoking status

	Non-smoker				Past smoker	Current smoker
	Without secondhand smoking	With secondhand smoking				
		Home	Other places	Home and other places		
Men						
Adjusted number of teeth^c^, mean (SD)	21.8 (7.02)	20.7 (7.01)	22.1 (7.01)	21.9 (7.01)	20.5 (7.00)	19.6 (7.02)^a^
Adjusted number of teeth^d^, mean (SD)	22.2 (6.92)	21.0 (6.91)	21.8 (6.80)	21.4 (6.95)	20.3 (6.81)	19.7 (6.82)^a^
Adjusted number of n-FTUs^c^, mean (SD)	6.34 (4.25)	6.22 (4.24)	6.84 (4.24)	6.83 (4.24)	5.88 (4.25)	4.82 (4.25)^a^
Adjusted number of n-FTUs^d^, mean (SD)	6.56 (4.18)	6.40 (4.17)	6.74 (4.10)	6.42 (4.19)	5.73 (4.11)	4.92 (4.12)^b^
Women						
Adjusted number of teeth^c^, mean (SD)	18.9 (6.96)	19.2 (6.93)	21.2 (6.93)	20.0 (6.94)	20.9 (6.95)	19.5 (6.94)
Adjusted number of teeth^d^, mean (SD)	18.9 (6.77)	19.4 (6.73)	21.4 (6.76)	19.7 (6.78)	19.7 (6.80)	19.1 (6.79)
Adjusted number of n-FTUs^c^, mean (SD)	4.63 (3.98)	4.57 (3.97)	5.57 (3.97)	5.20 (3.97)	4.88 (3.98)	3.73 (3.97)
Adjusted number of n-FTUs^d^, mean (SD)	4.62 (3.91)	4.66 (3.88)	5.39 (3.90)	5.05 (3.91)	4.18 (3.93)	3.46 (3.92)

^a^significantly different from nonsmoker without secondhand smoking (*p* < 0.05)

^b^significantly different from nonsmoker without secondhand smoking (*p* < 0.01)

^c^Adjusted for age

^d^Adjusted for age, education level, history of diabetes, BMI, alcohol consumption, perceived mental stress, presence of family dentist, and oral hygiene

As to women, the numbers of teeth or n-FTU in non-smokers with secondhand smoking ranged from 19.2 to 21.4, or 4.57 to 5.57, respectively. The numbers of teeth or n-FTUs in past smokers were 20.9 and 19.7, or 4.88 and 4.18, and those in current smokers were 19.5 and 19.1, or 3.73 and 3.46, respectively. There were no significant dentition differences from non-smokers without secondhand smoking in women.

## Discussion

In men, current smokers had a significantly higher risk of having severe periodontal disease and fewer teeth and n-FTUs than non-smokers without secondhand smoking. This result is consistent with that of previous studies which reported a significant association between active smoking and periodontal disease or dentition status [3–7]. About half of male smokers in this study were heavy drinkers, and several studies indicated that excessive consumption of alcohol was related with poor periodontal health [19, 20]. In addition, heavy drinkers might have poor health behaviors such as an inappropriate oral hygiene procedure, which could also negatively affect oral health.

An increased risk for periodontal disease or tooth loss was not observed among past smokers. Large variances in the period and length of smoking cessation among past smokers probably prevented detection of a significant risk. If the proportion of long-time quitters is large, for example, the influence of smoking will be diminished. Further, there may be potential differences in health behavior between quitters and current smokers. As for women, no significant effects of smoking on periodontal disease nor dentition were found. It may be probably due to the lack of statistical power because of the very small sample sizes in smokers.

Among male subjects, non-smokers with secondhand smoking only at home or both at home and other places showed a significantly higher prevalence of periodontal disease compared to nonsmokers without secondhand smoking, after controlling for other potential risk indicators for periodontal disease. This finding implies that the effect of secondhand smoking on the prevalence of periodontal disease occurs when nonsmokers are exposed to secondhand smoke, especially by their family members. It is plausible that higher exposure to secondhand smoke at home compromises periodontal health to a greater extent than lower exposures to secondhand smoke only at places other than home. An earlier study also demonstrated that exposure to secondhand smoke and periodontal disease among non-smokers had a dose-dependent relationship [12].

Non-smoking men with secondhand smoking showed similar risk for having periodontal disease to current smokers, and the OR of secondhand smoking in this study was a little higher compared to that reported in previous studies. However, it is difficult to simply compare current results with those in formerly conducted studies, because each study used different secondhand smoking criteria or assessment methods [10–14]. For instance, some studies used the hours per week and others used the frequency per day for exposure to smoke as criteria. In addition, because confounding factors such as age, gender, socio-economic status, stress and oral hygiene play a critical role in periodontal disease, consideration of these factors in analyzing the associations can change the results. Further, estimates of the periodontitis diagnosis vary extensively depending on case definition and the measurement protocol for periodontal disease [21].

Contrary to the results in men, no significant relationships were detected between secondhand smoking and periodontal disease in women. The mean age of women was almost the same as that of men. About 85 % of women lived with husband and 21 % were homemakers as of 1990. It is not clear why secondhand smoking was not associated with periodontal disease in women. However, some reasons are speculated. First, the actual amount of secondhand smoke might be smaller in female subjects who reported secondhand smoking. There is a study suggesting that despite women self-reported more exposure to secondhand smoke than men their actual serum cotinine values were lower [22]. Or women might tend to stay away from smokers at home to avoid being exposed to secondhand smoke for some reason including concern for health. It is reported that more women thought smoking was a risk factor for cancer than men [23].

There is also a research regarding gender related biological differences in nicotine metabolism, which shows a nicotine and cotinine clearances are higher in women than in men [24]. Further, a study demonstrated that smoking-related increase in intima-media thickness (IMT) was observed in men but not in women, suggesting a possible protection of women from structural arterial alteration of smoking [25]. The increased IMT was reported to be associated with poorer periodontal status [26]. These studies may imply that women might be less susceptible to the effects of smoke exposure than men physically and biologically. The other conceivable reason would be the influence of other confounding factors such as hormones for periodontal disease, and the possibility of findings provided by the artifact also cannot be denied.

Active and secondhand smoking may affect periodontal disease through similar mechanisms, but with a different magnitude. Non-smokers exposed to secondhand smoke absorb nearly one-third of the amount of nicotine per cigarette compared to that absorbed by current smokers [27]. Cigarette smoke affects periodontal disease locally and systemically [12]. Local effects include vasoconstriction caused by nicotine and decreased oxygen tension, which causes an enhancement of subgingival anaerobic bacteria colonization. A number of studies report that smoking has a detrimental effect on the subgingival microflora. Cotinine, enhances the potency of toxins produced by periodontopathogenic bacteria such as *Prevotella intermedia*, *Prevotella nigrescens*, *Treponema denticola*, and *Porphyromonas gingivalis*, which may enhance the progression of periodontal disease [28]. Recent research also finds that tobacco smoke and components alter the bacterial surface and promote biofilm formation in several periodontal related pathogens, including *Porphyromonas gingivalis* and *Aggregatibacter actinomycetemcomitans* [29].

Systemic effects include altered chemotaxis, phagocytosis of polymorphonuclear leukocytes, suppressed osteoblast proliferation, stimulated alkaline phosphatase activity and reduced antibody production [30]. The concentration of inflammatory markers, including interleukin-1β, lactoferrin, albumin and aspartate amino-transferase, in the saliva is reported to increase in subjects exposed to cigarette smoke [14, 31, 32]. The exposure of gingival epithelial cells to cigarette smoke results in a time-dependent loss of cell growth, which may occur through apoptotic and necrotic phenomena [33]. Further, cigarette smoke produces significant morphological and functional deregulation in gingival fibroblasts, predisposing an individual to oral infections [34].

In the present study, no significant associations were found between secondhand smoking and decreased number of teeth or n-FTUs. Although secondhand smoking had a harmful effect on periodontal heath, its effects were considered not strong enough to result in a significant loss of teeth and posterior occlusion. Of course, tooth loss can occur for reasons other than periodontal disease, e.g., by advanced dental caries. Therefore, the degree of influence of smoking on tooth loss by periodontal disease could be changed depending on the proportion of tooth loss caused by dental caries.

One of the limitations in this study is the reliability of self-reported smoking status, because the analysis using biomarkers such as cotinine was not conducted. It has been reported that smokers tend to underestimate tobacco use [35], and non-smokers also underestimate exposure to secondhand smoke [36]. Therefore, some misclassification regarding both active and secondhand smoking status could occur. Further, an individual’s lifetime changes in the location of home or workplace as well as in the environment (such as room ventilation, concentration of smoke and density of smokers) may complicate and make the estimate of actual exposure to secondhand smoke difficult. Use of more objective biomarkers such as cotinine, thiocyanate and carbon monoxide, may be preferable to self-reporting for measuring the amount of secondhand smoking, but the adoption of such biomarkers is not realistic in epidemiological studies. Another weakness of biomarkers like cotinine, is that, given an average 16 h half-life, cotinine levels reflect relatively short-term exposure to tobacco not long-term exposure [27].

Besides, many other confounders other than smoking status which includes oral health related behaviors may also have changed during the long study period. Therefore the findings in this study should be interpreted with caution because taking into account these variables into the analysis may change the results.

The current case definition of periodontal disease was made based only on the measurement of probing pocket depth. Measurements by both probing pocket depth and clinical attachment level would evaluate periodontal status more precisely. Inter- or intra-reliability tests were not calculated in this study because more than 40 dentists participated in the study. Therefore, the individual difference in clinical examination criteria may potentially influence on the results of oral health status. In addition to these limitations, a conflicting result between men and women demands a longitudinal study with strict smoking exposure estimates and periodontal disease definition to further investigate the role of active and secondhand smoking on oral health.

Nonetheless, the current study indicated a possible relationship of active smoking with periodontal disease and tooth loss as well as of secondhand smoking with periodontal disease in men. The effects of secondhand smoking on periodontal disease were observed when non-smokers were exposed to secondhand smoke at home. Even for smokers, the risk for periodontal disease is increased more if exposed to secondhand smoke. Higher risk for oral disease among smokers and non-smokers with secondhand smoking suggests that it is necessary to provide smoking cessation measures for smokers and to raise awareness of the risk of secondhand smoking for both smokers and non-smokers. Active and passive smoking has harmful effects not only on oral health but also on systemic health. Because manifestations of smoking, such as tooth pigmentation, are relatively easily found by clinical dental examination, oral health professionals should play a crucial role in providing health education and recommending smoking cessation to their patients.

## Conclusions

Active smoking was suggested to be associated with a higher risk of severe periodontal disease and tooth loss, and secondhand smoking with periodontal disease in men. Both active and secondhand smoking are preventable health threats in the society. The present study indicates that it is imperative for health and oral health professionals to enlighten people about the harmful effects of smoking, not only on their own health but also on others’ health.
